# Long Term Outcomes and Effects of Surgery on Degenerative Spinal Deformity: A 14-Year National Cohort Study

**DOI:** 10.3390/jcm8040483

**Published:** 2019-04-10

**Authors:** Yu-Chun Chen, Wen-Cheng Huang, Hsuan-Kan Chang, Jiing-Feng Lirng, Jau-Ching Wu

**Affiliations:** 1 School of Medicine, National Yang-Ming University, Taipei 11221, Taiwan; yuchn.chen@gmail.com (Y.-C.C.); wchuang@vghtpe.gov.tw (W.-C.H.); hsuankanchang@gmail.com (H.-K.C.); jflirng@vghtpe.gov.tw (J.-F.L.); 2 Department of Biomedical Engineering, School of Biomedical Science and Engineering, National Yang-Ming University, Taipei 11221, Taiwan; 3 Department of Family Medicine, Taipei Veterans General Hospital, Taipei 11217, Taiwan; 4 Institute of Hospital and Health Care Administration, National Yang-Ming University, Taipei 11221, Taiwan; 5 Department of Neurosurgery, Neurological Institute, Taipei Veterans General Hospital, Taipei 11217, Taiwan; 6 Department of Biomedical Imaging and Radiological Sciences, National Yang-Ming University, Taipei 11221, Taiwan; 7 Department of Radiology, Taipei Veterans General Hospital, Taipei 11217, Taiwan

**Keywords:** Degenerative Spinal Deformity (DSD), mortality, respiratory problems, hip fractures, surgery

## Abstract

Degenerative spinal deformity (DSD) has become a prevalent cause of disability and pain among the aging population worldwide. Though surgery has emerged as a promising option for DSD, the natural course, outcomes, and effects of surgery on DSD have remained elusive. This cohort study used a national database to comprehensively follow up patients of DSD for all-cause mortality, respiratory problems, and hip fracture-related hospitalizations. All patients were grouped into an operation or a non-operation group for comparison. An adjustment of demographics, comorbidities, and propensity-score matching was conducted to ameliorate confounders. A Cox regression hazard ratio (HR) model and Kaplan-Meier analysis were also applied. The study comprised 21,810 DSD patients, including 12,544 of the operation group and 9266 of the non-operation group. During the 14 years (total 109,591.2 person-years) of follow-up, the operation group had lower mortality (crude hazard ratio = 0.40), lower respiratory problems (cHR = 0.45), and lower hip fractures (cHR = 0.63) than the non-operation group (all *p* < 0.001). After adjustment, the risks for mortality and respiratory problems remained lower (adjusted HR = 0.60 and 0.65, both *p* < 0.001) in the operation than the non-operation group, while hip fractures were indifferent (aHR = 1.08, *p* > 0.05). Therefore, surgery for DSD is invaluable since it could reduce the risks of mortality and of hospitalization for respiratory problems.

## 1. Introduction

Degenerative Spinal Deformity (DSD) has become a prevalent cause of disability and pain among the aging population worldwide. The major medical comorbidities of DSD include pulmonary, cardiovascular, and rheumatic complications [1,2], which could cause a significant reduction in quality of life, hindrance in ambulation, and even mortality. Management of DSD includes medical or surgical strategies for symptom relief or scoliosis correction, all of which require individualized considerations. 

Surgical approaches for DSD have gained increasing popularity in modern spine care since various surgical techniques and instruments have demonstrated their effectiveness in the relief of clinical symptoms, amelioration of neurological deficits, and an increase of quality of life for patients with DSD [3,4,5,6,7]. However, there is a paucity of data on the natural course of DSD, medical comorbidities, and the long-term influences on quality of life in these patients. Though it is intuitive to infer that restoration of sagittal balance and correction of scoliosis are correlated with improvement in general health, it remains uncertain whether or not the spinal surgery for DSD truly attenuates the long-term risks of medical comorbidities or even mortality for these debilitated patients. 

There are various surgical and medical strategies which have been developed for corrective and palliative treatment for DSD. Some of the corrective surgery [8], including pedicle subtraction osteotomy [9], vertebral column resection [10], and anterior column release [11,12], could yield a remarkable restoration of spinal alignment. However, these major operations are not free of complications or morbidities [13,14,15], and some of them might jeopardize not only the quality of life but also the survival of these patients of DSD. The balance between maximization of the effects of treatment and minimization of the risks of morbidities would likely continue to evolve as the technologies advance. 

This study aimed to compare patients of DSD who were managed differently, applying medical versus surgical strategies, in a national cohort. The universal coverage of the government-sponsored health insurance system allowed comprehensive follow-up of every DSD patient enrolled, which was a unique opportunity for the evaluation of the natural course of the disease, for medical comorbidities, and the effectiveness of surgical treatment. To date, this was the largest cohort of DSD patients with the most extensive follow-up, focusing on the prediction of outcomes.

## 2. Materials and Methods

### 2.1. Data Source and Ethical Concerns

The present study adapted an open claim database of Taiwan, the National Health Insurance Research Database (NHIRD). The database was derived from the National Health Insurance (NHI) program, which was launched by the government of Taiwan in 1995, aimed at providing unrestricted access to all contracted medical care services and universal health insurance for all residents in Taiwan. To date, the NHI program has comprehensively covered 99% of the Taiwanese population and contracted with more than 97% of the providers of healthcare services in Taiwan. After cross-checking for validity, de-identification, and anonymization, the claim data of the NHI program are released publicly for research purposes. The NHIRD contains comprehensive information on each of the insured subjects including gender, date of birth, dates of clinical visits (both preventive services and emergent visits) and hospitalization, the International Classification of Diseases (Ninth Revision) Clinical Modification (ICD-9-CM) codes of diagnoses, ICD codes of surgical procedures, etc.

The protocol of the present study was approved by the Institutional Review Board of Taipei Veterans General Hospital (IRB# 2018-09-0006CC). Since all identifying personal information in the NHIRD is encrypted, the Institutional Review Board waived the requirement for written informed consent from each of the patients involved. 

### 2.2. Identification of Study Cohort

This was a population-based retrospective cohort study focusing on elderly patients who were first hospitalized for spinal deformity. We enrolled all patients aged >50 years who had been first hospitalized for spinal deformity (ICD-9-CM: 737.x or 738.5) between 1 January 2000 and 31 December 2013. To ensure the validity of time of first hospitalization (index hospitalization), we recruited patients who were hospitalized (not clinic visits) for spinal deformity as the main reason of the index hospitalization by limiting spinal deformity as the main diagnosis code (first 3 out of 5 diagnoses codes of each record). Moreover, we traced back for at least five years for confirmation. Any patient who had any previous hospitalization for any spinal problems was excluded from analysis. To ensure the accuracy of inclusion, only DSD patients, rather than those with other minor spinal problems, were included. Thus, there was minimal selection bias because only elderly patients with similar severity of spinal deformity that required hospitalization were enrolled as our study cohort. In other words, patients with various minor severities of degenerative spinal problems, such as simple disc herniation that required discectomy surgery, were excluded.

**Figure 1:**
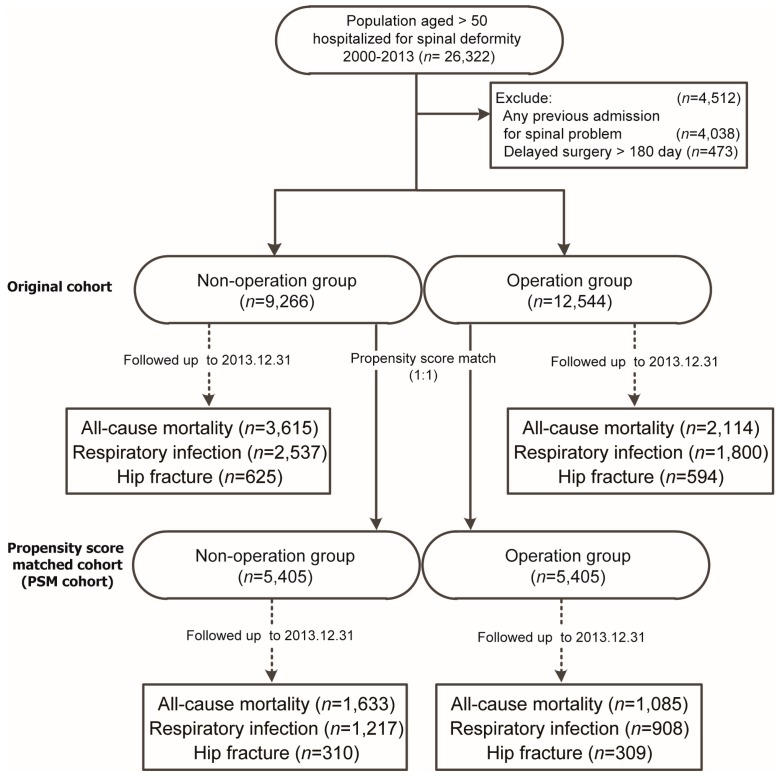
Flowchart of data processing for outcomes of a cohort with degenerative spinal deformity (DSD), with a subgroup analysis of patients from both groups and who were matched with propensity scores. DSD patients in Taiwan, 2000–2013 (*n* = 26,322).

Any discharge from the hospitalization without surgery for more than 180 days afterwards was excluded for analysis, whereas only patients who had surgery within the 180 days would be included for analysis (Figure 1).

### 2.3. Treatment Strategies: Operation Group versus Non-Operation Group

To evaluate the outcomes of operation management, all patients were assigned to either the operation group or the non-operation group according to whether they received surgery or not during or within 180 days of their index hospitalizations. The surgery for DSD was determined by receiving any or combined anterior, lateral, posterior, or lumbo-sacral fusion surgery, including all ICD-9 procedure codes: 81.04, 81.05, 81.06, 81.07, and 81.08. Patients who had undergone the lumbar spine fusion surgery were then assigned to the operation group according to the records of the procedures during hospitalization. Theoretically, all patients of DSD who underwent surgery were thus identified and enrolled, even including a small number of patients who postponed their surgery to their next hospitalization (less than 180 days) in the operation group. However, a few patients who received such surgery later than 180 days from the indexed hospitalization were excluded. The rest of the DSD patients in the study cohort did not undergo any lumbar spinal fusion surgery and were assigned to the non-operation group.

The information bias (recall bias) and misclassification bias (e.g., error in assigning operated patients to the non-operation group or vice versa) were both very minimal in the current study since the database enabled us to follow-up patients in every hospital in Taiwan with a very minimal rate of loss to follow-up. 

### 2.4. Follow-Up Outcomes 

The most critical health-related subsequent events or outcomes of DSD in the elderly population were mortality, respiratory problems, and disability related to difficulty in ambulation. The current study was thus designed to specifically follow-up these outcomes of DSD. Hospitalization for impairment of lung function, decreased functional capacity, respiratory tract infections, hip fractures, and all-cause mortality rates were followed-up closely for patients of DSD. Since the NHIRD uniquely provided a very comprehensive follow-up of the entire cohort, all patients in the study were followed up to the end of this study period with a very minimal loss to follow-up. We used the discharge codes as the reason for admission and followed-up the first occurrence of the following events: Admission for respiratory infections or hip fractures. Furthermore, any admission and re-operations within 180 days after the indexed surgery would be tracked, even if they were in different institutes, departments (e.g., orthopedics or neurosurgery), or physically distant, owing to the universal coverage of the government supported monopolistic system of health insurance. In addition, because all medical care providers are contracted with the BNHI, which allows all patients unrestricted access, any post-operative events would very likely be captured by the NHIRD. Due to the rigorously monitored billing processes by the BNHI, admissions of these patients would have little chance of loss to follow-up.

### 2.5. Demographics, Medical Comorbidities, and Other Covariates

Covariates of the study were adjusted for comparison. To evaluate the effects on mortality and admission for respiratory infections and hip fractures after the index operation for DSD, the study included and controlled the potential risk factors, including age, sex, and other identifiable risk factors, in a multivariate analysis. We categorized and included the most prevalent co-morbidities according to Elixhauser’s comorbidity model. Patients’ co-morbidities were determined by the presence of either diagnostic codes in the outpatient records or discharge codes in the database within two years before the date of the index date [16,17]. A total of 10 kinds of medical co-morbidities of the highest prevalence rates (>5%) were examined as covariates. These medical co-morbidities included anemia, chronic liver disease, chronic pulmonary disease, congestive heart failure, diabetes, fluid and electrolyte disorders, hypertension, rheumatoid arthritis/collagen diseases, tumor(s), and valvular disease (Appendix A Table A1).

### 2.6. Identification of Propensity Score Matched Cohort

To evaluate treatment effects of surgical intervention and to minimize the confounder effects that were contributed by factors that did not exist in our database (such as functional status), a one-to-one matched comparison between the operation and non-operation groups was derived from the original cohort, based on a propensity score calculated by age, sex, and medical comorbidities [18]. 

As the propensity score represents the probability for patients allocated into the group, the authors deliberately picked patients from the operation group and non-operation group with close matches on propensity scores by setting a very low threshold for matching (matching caliper = 0.0001). There was an inherent bias for DSD patients who were sicker or had more co-morbidities to opt for non-operation treatment and for healthier patients to opt for operation treatment; the propensity score matched cohort analysis had a tight matching caliper that led to a greatly reduced treatment-assignment bias [19].

### 2.7. Statistical Analysis

All the data were linked using the SQL server 2017 (Microsoft Corp, Redmond, WA, USA) and analysed by Stata software (Stata Corp, College Station, TX, USA). The Kaplan-Meier method and a log-rank test were used to estimate and compare cumulative rates between the operation and non-operation groups. Adjusted hazard ratios (aHR) and a 95% confidence interval (95% C.I.) for subsequent hospitalizations, including those for respiratory problems (e.g., pneumonia) and hip fractures, were estimated using a Cox proportional regression model. To reduce misclassification, propensity score matching was adapted and opted for very low thresholds for matching tolerance (<0.0001) for adjustment for both measurable and unmeasurable confounders. Furthermore, competing risk survival analyses were adapted for adjustment of respiratory problems (e.g., pneumonia) and hip fracture related influences. For the relatively rare outcomes, a bootstrap method with 1000 repeats re-sampling was adapted to obtain a less-biased estimation of aHR, with standard error (S.E.) of adjusted hazard ratio and P-value reported instead of a conventional 95% C.I. [20]. A two-tailed level of 0.05 was considered statistically significant.

### 2.8. Quantitative Bias Analysis

Probabilistic sensitivity analysis was conducted to minimize bias of imprecise or inaccurate diagnostic coding of DSD. Though the coding of the NHIRD has been validated [21], we postulated that there was some upcoding of DSD; thus, a number of patients were admitted for their neurological complaints (e.g., spinal root problems or cauda equina syndrome) other than mal-aligned spine (DSD), and we conducted a confirmation examination. We modelled such a condition (up-coding) as an unmeasurable confounder which could lead to distortion of the association between the operation and outcomes. To minimize such a confounder bias, probabilistic sensitivity analysis was conducted using R 3.5.1 (The R Foundation for Statistical Computing) for evaluation of the influences of up-coding behaviour [22]. A confounder bias corrected risk ratio (bcRR) was calculated by assuming that a high prevalence of up-coded DSD follows a uniform distribution ranging from 50% to 90% (i.e., at least 50% of hospitalizations might be over-coded as DSD, which was a very conservative assumption). A simulated 95% limit of bcRR was calculated using 100,000 repeats. The bcRR was compared to observed incidence rate ratios to quantify the effect of bias.

Importantly, the coding in NHIRD also serves for billing and social welfare benefits; thereby, it is subject to prudent internal monitoring by the BNHI of Taiwan. Fraudulent coding is heavily penalized, and, therefore, such monitoring promotes accuracy of coding. Hence, the number of DSD patients in the current study should be valid.

## 3. Results

### 3.1. Overall Outcomes of DSD

The overall outcome of DSD is debilitating, and surgery could partially reverse the deterioration. There were a total of 21,810 patients in the entire DSD cohort, which yielded an overall follow-up of 109,591.2 person-years. The overall mortality rate, including both operation and non-operation groups of patients, was 51.3 per 1000 person-years since the hospitalization. In addition, the rates of admission for respiratory problems and hip fracture were 43.7 and 11.5 per 1,000 person-years, respectively.

The entire cohort of DSD was divided into the operation and non-operation groups and followed-up for the above-mentioned outcomes till the end of the study, which was 31st December 2013 (Figure 1).

### 3.2. Treatment Strategies versus Outcomes

The operation group was significantly different to the non-operation group in gender, age, co-morbidities, and outcomes in the original cohort (Table 1). After matching with the propensity score model, the two groups were quite similar in gender, age, and medical co-morbidities. In addition, the operation group had better outcomes, including less mortality and respiratory problems, than the non-operation group, in the matched cohort comparison.

In the original cohort, the operation group had less risk of all-cause mortality (crude hazard ratio = 0.40, and adjusted HR = 0.60, both *p* < 0.001), less respiratory problems (cHR = 0.45, and aHR = 0.65, both *p* < 0.001), and less hip fractures (cHR = 0.63, *p* < 0.01, and aHR = 1.08, *p* > 0.05) than the non-operation group. Moreover, in the propensity score matched cohort, the operation group had less risk for all-cause mortality (cHR = 0.64, aHR = 0.66, both *p* < 0.001) and less respiratory problems (cHR = 0.68, aHR = 0.74, both *p* < 0.001), but similar hip fractures (cHR = 0.95, aHR = 1.07, both *p* > 0.05), than the non-operation group (Table 2).

**Figure 2:**
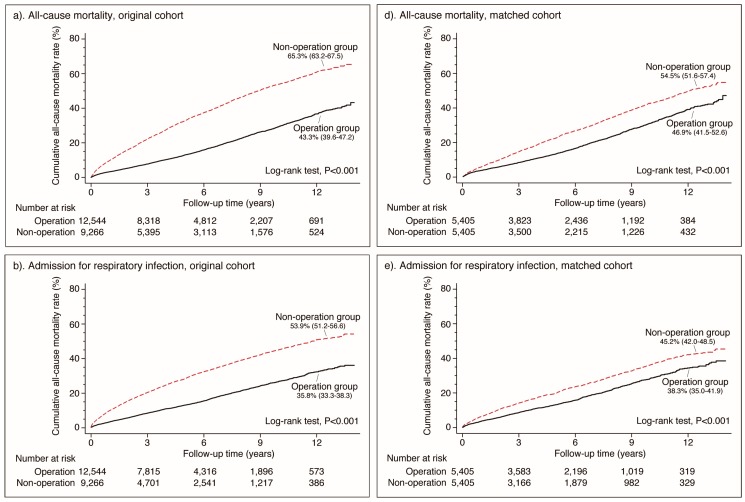

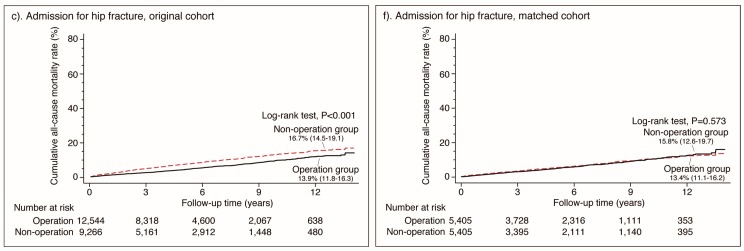
Cumulative incidences of all-cause mortality (**a**,**d**), respiratory problems (**b**,**e**), and hip fractures (**c**,**f**) during the 14 years of follow-up of both the non-operation and operation groups.

Furthermore, in the survivor analysis of the original cohort, the operation group had less accumulative incidences of mortality, respiratory problems, and hip fractures than the non-operation group (Figure 2). In the propensity score matched cohort, the operation group also had less accumulative incidences of mortality and respiratory problems, but similar hip fractures, than the non-operation group.

### 3.3. Probabilistic Sensitivity Analysis

The influences caused by the unmeasurable confounders (up-coded DSD) minimally affected the results of the current study. The bcRR for all-cause mortality, admission for respiratory infection and admission for hip fracture were similar to the observed incidence rate ratio in either the original cohort or the propensity score matched cohort (Table 3) and thus suggested minimal effects of bias on the current results.

## 4. Discussion

Tremendous advancements in the past several decades have been made in medicine to improve patients’ survival and quality of life. There also have been various strategies tailored for patients with DSD, aimed at enhancing ambulation, increasing survival, and mitigating complications.

The present study adapted a national scale cohort of DSD patients (*n* = 21,810) with an observation span of 14 years to investigate the long-term outcomes, including major complications and mortalities. These DSD patients were divided into the operation group and the non-operation group (12,544 and 9266 patients, respectively) and followed-up for subsequent re-admissions for respiratory problems, hip fractures, and overall survival. During the total follow-up of 109,591.2 person-years, the operation group had lower risks of mortality (crude hazard ratio = 0.40), respiratory problems (cHR = 0.45), and hip fractures (cHR = 0.63) than the non-operation group (all *p* < 0.001). After adjustments, which were made for demographics and all the other medical co-morbidities, the operation group still had lower risks for mortality and respiratory problems (adjusted HR = 0.60 and 0.65, both *p* < 0.001) than the non-operation group, while hip fractures were insignificantly different (aHR = 1.08, *p* > 0.05). Therefore, surgical treatment for DSD is invaluable among other management strategies, since surgery could reduce the risks of mortality and hospitalization for respiratory problems. 

This was the first study to shed light on the natural course of DSD and moreover to document the long-term benefits of surgery in the management of DSD. The improvement of survival and complications demonstrated by the national cohort in the comprehensive follow-up warranted surgery for DSD in selected patients. 

Past studies have demonstrated that DSD was associated with increased mortality in older men and women [23,24,25]. Though osteoporotic vertebral fracture and associated disability could be correlated with medical complications and therefore increased mortality in patients of DSD, in a prospective cohort of 610 women, increased kyphosis was an independent risk factor for all-cause mortality [25]. On the other hand, other multiple reports addressed the morbidity and mortality of surgery for correction of DSD [26,27]. Various studies have demonstrated that surgery could enhance the quality of life through the correction of DSD and improve sagittal balance [28,29,30]. Nevertheless, there was a paucity of evidence on whether surgical management of DSD could minimize morbidities and prolong survival despite the advancement of surgical techniques and approaches made in the past decade [8,31,32,33]. The above mentioned studies, albeit focused on DSD, lacked a longitudinal observation period or high enough follow-up rates to capture the long-term outcomes. The subsequent hospitalizations for pneumonia, pulmonary problems, major disability (i.e., hip fractures), and eventually mortality were consequences of severe DSD that required years of observation. It was difficult to investigate by clinical series, even in the busiest spine centers. Therefore, the present study took advantage of the NHI database with its universal and monopolistic coverage. The cohort also allowed comparison of surgical and medical management, and thus provided a unique opportunity of identification of the efficacy of treatment. The present study demonstrated a remarkably lower risk of mortality and respiratory problems (adjusted HR = 0.60 and 0.65) for the patients who underwent surgery than those who did not. The results indicated that 35%–40% less respiratory problems and lower mortality could be achieved by surgery for DSD patients.

Surgical approaches for DSD have rapidly evolved over the years. There were multiple emerging techniques of minimally invasive surgery (MIS) for correction of DSD, including anterior, lateral, posterior, and hybrid approaches [8,32,33,34,35]. The evolution of techniques and principles of MIS have led to significant advances in the field aimed at reducing soft-tissue trauma and mitigating surgery-related morbidities. Using these MIS technologies, the surgery for DSD has translated into more rapid recovery, lower infection rates, and higher cost savings when compared to conventional surgery [36]. Though the advances have not been proven to be superior than traditional surgery for mortalities in DSD, the present study successfully proved the long-term benefit of survivorship. It is reasonable to infer that, with these advances in technology, the surgical outcomes of DSD could surpass non-operation management. However, future investigations are required to corroborate the theory. The present study, using data extracted from the NHI database, did not include detailed surgical approaches and did not distinguish the traditional open surgery from modern percutaneous placement of pedicle screws for stabilization. However, there was marked improvements in the rates of respiratory problems, all-cause mortality, and, in the original cohort rather than the propensity score matched cohort, hip fractures.

The NHIRD did not incorporate operation notes for analysis of the exact anatomic level, type, and degree of DSD. Detailed pre- and post-operation neurologic examinations of each patient were also not available for analysis. It was also impossible to trace back through each individual’s medical charts for the exact fusion levels, types of instrumentation, and fusion status. However, the identification of surgery in the cohort analysis was extremely accurate because the NHIRD underwent vigorous internal control and checking. This record was initially used for billing and social welfare benefits, and, therefore, it was and is under prudent internal monitoring so that fraudulent coding would be heavily penalized, which promotes accuracy of coding. Hence, the number of surgeries performed in the cohort was accurate, and the comparison of the two groups would have minimal bias [37,38,39,40]. Prediction of a disease’s natural course and investigation for long term outcomes, such as mortality, are the perfect application of the NHIRD. 

Regarding the inclusion criteria of the current study of DSD, combining age >50 years and hospitalization for ICD-9 of 737.x or 738.5, under the strict internal control of the National Health Insurance of Taiwan for billing and social welfare, enforced by law, it should very much truly reflect the reality of clinical management of DSD in Taiwan. Since the government-supported NHI also allows DSD patients to undergo in-house rehabilitation treatment, many of the DSD patients enrolled in the current study were likely hospitalized for physical therapy, rehabilitation, or medical treatment (e.g., acupuncture, injection, shock-wave, universally covered by the NHI), as the non-operative group. Nonetheless, the diagnostic- and procedure-codes based inclusion were different from the commonly reported patients of DSD whose diagnoses were usually based on radiographs [13,14,15], reported in the literature. 

There were limitations to the study. The cohort study did not investigate the detailed operation notes and thus could not address the types of instrumentation, fusion levels, degree of correction for DSD, pseudarthrosis, and the extent of involvement of MIS technologies. However, these corrective surgeries of DSD, which commonly used many screws, grafting materials and instrumentation, were usually prudently reviewed by peer-groups of spinal surgeons, usually from either neurosurgery or orthopedics. Moreover, there could be selection bias in that patients who opted for surgery or those who had been suggested for surgery might be healthier and would have less complications and better longer-term outcomes, even without surgery. Nevertheless, the differences between the two groups were insignificant and matched by propensity scores, and the medical co-morbidities were adjusted with the statistics (Table 1 and Table 2). Since the major long-term outcomes, including respiratory problems and survival, were better for the group of patients who had surgery for DSD, this study advocates surgery for selected patients with DSD.

## 5. Conclusions

In this national cohort of DSD patients over 14 years, the operation group had lower risks of mortality, respiratory problems, and hip fractures than the non-operation group. Even after adjustments made for demographics and other medical co-morbidities, the operation group still had lower risks for mortality and respiratory problems (adjusted HR = 0.60 and 0.65) than the non-operation group. Therefore, surgical treatment for DSD is invaluable among other management strategies, since surgery could reduce the risks of mortality and hospitalization for respiratory problems. 

## Figures and Tables

**Table 1:** Demographics, medical co-morbidities, and long-term follow-up outcomes between the non-operation group and operation group in the original cohort (aged >50, *n* = 21,810) and the propensity score matched cohort (*n* = 10,810) in Taiwan, 2000–2013.

	Original Cohort *n* = 21,810		*P*-Value	Propensity Score Matched Cohort ^1^ *n* = 10,810		*P*-Value
	Non-Operation Group	Operation Group		Non-Operation Group	Operation Group	
	*n* = 9266 (%)	*n* = 12,544 (%)		*n* = 5405 (%)	*n* = 5405 (%)	
Gender			<0.001			1.000
Female	6005 (64.8)	8564 (68.3)		3524 (65.2)	3524 (65.2)	
Male	3261 (35.2)	3980 (31.7)		1881 (34.8)	1881 (34.8)	
Age mean (SD)	72.4 (11.0)	68.0 (8.4)	<0.001	69.2 (9.5)	69.2 (9.5)	1.000
Co-morbidities						
Anemia	1021 (11.0)	824 (6.6)	<0.001	148 (2.7)	148 (2.7)	1.000
Chronic liver disease	991 (10.7)	1601 (12.8)	<0.001	272 (5.0)	272 (5.0)	1.000
Chronic pulmonary disease	2103 (22.7)	2112 (16.8)	<0.001	569 (10.5)	569 (10.5)	1.000
Congestive heart failure	797 (8.6)	655 (5.2)	<0.001	93 (1.7)	93 (1.7)	1.000
Diabetes	1597 (17.2)	2934 (23.4)	<0.001	677 (12.5)	677 (12.5)	1.000
Fluid and electrolyte disorders	893 (9.6)	622 (5.0)	<0.001	117 (2.2)	117 (2.2)	1.000
Hypertension	3132 (33.8)	5031 (40.1)	<0.001	1549 (28.7)	1549 (28.7)	1.000
Rheumatoid arthritis/collagen diseases	549 (5.9)	1031 (8.2)	<0.001	141 (2.6)	141 (2.6)	1.000
Tumor	728 (7.9)	557 (4.4)	<0.001	104 (1.9)	104 (1.9)	1.000
Valvular disease	620 (6.7)	762 (6.1)	0.065	108 (2.0)	108 (2.0)	1.000
Outcome						
All-cause mortality	3615 (39.0)	2114 (16.9)	<0.001	1633 (30.2)	1085 (20.1)	<0.001
Admission for respiratory infection						
Post-OP admission rate	2537 (27.4)	1800 (14.3)	<0.001	1217 (22.5)	908 (16.8)	<0.001
Admission for hip fracture						
Post-OP admission rate	625 (6.7)	594 (4.7)	<0.001	310 (5.7)	309 (5.7)	0.967

^1^ Propensity scores were calculated using multiple logistic regression, with sex, gender, and 10 co-morbidities listed. Patients in the operation and non-operation groups were matched closely with a very low matching tolerance (matching caliber = 0.0001) to evaluate treatment effect of the operation for spinal deformity.

**Table 2:** Follow-up for all-cause re-admissions, admission for respiratory infection and admission for hip fracture after the index admission for spinal deformity in the original cohort (aged > 50, *n* = 21,810) and propensity score matched cohort (*n* = 10,810) in Taiwan, 2000–2013.

Follow-Up after Index Spinal Deformity Admission	Original Cohort *n* = 21,810		Propensity Score Matched Cohort ^1^ *n* = 10,810	
	Non-Operation Group	Operation Group	Non-Operation Group	Operation Group
All-cause mortality				
Incidence of all-cause mortality (per 1000 person-years)	80.1	32.3	54.3	34.9
Number of mortality	3548	2111	1615	1083
Observed person-years	44,313.0	65,278.2	29,719.0	30,991.2
Crude hazard ratio (95% C.I.)	1.00	0.40 (0.38–0.43) ***^,3^	1.00	0.64 (0.59–0.69) ***
Adjusted hazard ratio (95% C.I.) ^2^	1.00	0.60 (0.57–0.64) ***	1.00	0.66 (0.61–0.72) ***
Admission for respiratory infection				
Incidence of admission (per 1000 person-years)	66.1	29.5	46.0	31.5
Number of occurrences	2537	1794	1217	905
Observed person-years	38391.1	60,714.2	26,444.2	28,716.8
Crude hazard ratio (95% C.I.)	1.00	0.45 (0.42–0.48) ***	1.00	0.68 (0.63–0.75) ***
Adjusted hazard ratio (95% C.I.) ^4^	1.00	0.65 (0.61–0.69) ***	1.00	0.74 (0.68–81.2) ***
Admission for hip fracture				
Incidence of hip fracture (per 1000 person-years)	14.8	9.3	10.8	10.3
Number of occurrences	625	593	310	309
Observed person-years	42,371.6	63,455.8	28,674.2	29,981.4
Crude hazard ratio (95% C.I.)	1.00	0.63 (0.57–0.71) ***	1.00	0.95 (0.81–1.12)
Adjusted hazard ratio (95% C.I.) ^4^	1.00	1.08 (0.95–1.22)	1.00	1.07 (0.91–1.25)

^1^ Propensity scores were calculated using multiple logistic regression, with sex, gender, and 10 co-morbidities. Patients in the operation and non-operation groups were matched closely with a very low matching tolerance (matching caliber = 0.0001) to evaluate the treatment effect of the operation for spinal deformity; the ^2^ adjusted hazard ratio was estimated using a Cox proportional hazard model controlling for age, gender, anemia, chronic liver disease, chronic pulmonary disease, congestive heart failure, diabetes, fluid and electrolyte disorders, hypertension, rheumatoid arthritis/collagen diseases, tumor(s), and valvular disease; ^3^ significance level: ***, *p* < 0.001; the ^4^ adjusted hazard ratio was estimated using a competing-mortality survival model controlling for age, gender, and co-morbidities in considering potential admission impeded by death.

**Table 3:** Observed incidence rate ratio and bias corrected rate ratio (bcRR) for outcomes after index spinal deformity admission in the original cohort (aged > 50, *n* = 21,810) and the propensity score matched cohort (*n* = 10,810) in Taiwan, 2000–2013.

Follow-Up after Index Spinal Deformity Admission	Original Cohort *n* = 21,810	Propensity Score Matched Cohort ^1^ *n* = 10,810
All-cause mortality		
Observed incidence rate ratio (95% C.I.)	0.40 (0.38–0.43)	0.64 (0.59–0.69)
Bias corrected rate ratio (bcRR) (95% limit) ^2^	0.40 (0.37–0.44)	0.64 (0.58–0.71)
Admission for respiratory infection		
Observed incidence rate ratio (95% C.I.)	0.45 (0.42–0.48)	0.68 (0.63–0.75)
Bias corrected rate ratio (bcRR) (95% limit) ^2^	0.45 (0.41–0.49)	0.68 (0.62–0.76)
Admission for hip fracture		
Observed incidence rate ratio (95% C.I.)	0.63 (0.56–0.71)	0.95 (0.81–1.12)
Bias corrected rate ratio (bcRR) (95% limit) ^2^	0.63 (0.96–0.72)	0.95 (0.80–1.13)

^1^ Propensity scores were calculated using a multiple logistic regression with sex, gender, and ten co-morbidities. Patients in the operative and non-operative groups matched closely with a very low matching tolerance (matching caliber = 0.0001) to evaluate the treatment effects of operation for degenerative spinal deformity. ^2^ A confounder bias corrected risk ratio (bcRR) was calculated by assuming the prevalence of up-coded DSD follows a uniform distribution ranging from 50% to 90% (i.e., at least 50% of hospitalization might be over-coded as DSD, which was a very conservative assumption). A simulated 95% limit of bcRR was calculated using 100,000 repeats.

## Appendix A

**Table A1:** ICD-9-CM Coding Algorithms for Comorbidities.

Co-Morbidities	ICD-9-CM Codes
Depression	300.4, 301.12, 309.0, 309.1, 311
Psychoses	295.x–298.x, 299.1
Diabetes	(uncomplicated) 490x–492.x, 493.x, 494x-505.x, 506.4 (complicated) 250.0–250.3, 648.0
Hypertension	(uncomplicated) 401.1, 401.9, 642.0 (complicated) 401.0, 402.x–405.x, 642.1, 642.2, 642.7, 642.9
Cerebrovascular disease	362.34, 430.x–438.x
Paralysis	342.x–344.x, 438.2–438.5
Other neurological disorders	330.x–331.x, 332.0, 333.4, 333.5, 334.x, 335.x, 340, 341.1–341.9, 345.x, 347.x, 780.3, 784.3
Congestive heart failure	398.91, 402.01, 402.91, 404.01, 404.11, 404.13, 404.93, 428.x, 402.11, 404.03, 404.91
Valvular disease	093.2, 394.x–397.1, 397.9, 424.x, 746.3–746.6, V42.2, V43.3
Chronic pulmonary disease	490x–492.x, 493.x, 494x–505.x, 506.4
Liver disease	070.22, 070.23, 070.32, 070.33, 070.44, 070.54, 456.0, 456.1, 456.20, 571.0, 571.2–571.9, 572.3, 572.8, V42.7
Peptic ulcer disease excluding bleeding	531.41, 531.51, 531.61, 531.7, 531.91, 532.41, 532.51, 532.61, 532.7, 532.91, 533.41, 533.51, 533.61, 533.7, 533.91, 534.41, 534.51, 534.61, 534.7, 534.91
Peripheral vascular disorders	440.x, 441.x, 442.x, 443.1–443.9, 447.1, 557.1, 557.9, V43.4
Deficiency anemia	280.1–281.9, 285.2, 285.9
Rheumatoid arthritis/collagen vascular diseases	701.0, 710.x, 714.x, 720.x, 725.x

Source: [16,17,41,42].

## References

[1] Yagi M., King A.B., Boachie-Adjei O. (2011). Characterization of osteopenia/osteoporosis in adult scoliosis: Does bone density affect surgical outcome?. Spine.

[2] Xu L., Sun X., Huang S., Zhu Z., Qiao J., Zhu F., Mao S., Ding Y., Qiu Y. (2013). Degenerative lumbar scoliosis in Chinese Han population: Prevalence and relationship to age, gender, bone mineral density, and body mass index. Eur. Spine J..

[3] Yadla S., Maltenfort M.G., Ratliff J.K., Harrop J.S. (2010). Adult scoliosis surgery outcomes: A systematic review. Neurosurg. Focus.

[4] Wang M.Y., Mummaneni P.V. (2010). Minimally invasive surgery for thoracolumbar spinal deformity: Initial clinical experience with clinical and radiographic outcomes. Neurosurg. Focus.

[5] Dorward I.G., Lenke L.G. (2010). Osteotomies in the posterior-only treatment of complex adult spinal deformity: A comparative review. Neurosurg. Focus.

[6] Upadhyaya C.D., Starr P.A., Mummaneni P.V. (2010). Spinal deformity and Parkinson disease: A treatment algorithm. Neurosurg. Focus.

[7] Lenke L.G., Sides B.A., Koester L.A., Hensley M., Blanke K.M. (2010). Vertebral column resection for the treatment of severe spinal deformity. Clin. Orthop. Relat. Res..

[8] Park P., Fu K.M., Mummaneni P.V., Uribe J.S., Wang M.Y., Tran S., Kanter A.S., Nunley P.D., Okonkwo D.O., Shaffrey C.I. (2018). The impact of age on surgical goals for spinopelvic alignment in minimally invasive surgery for adult spinal deformity. J. Neurosurg. Spine.

[9] Wang M.Y., Bordon G. (2016). Mini-open pedicle subtraction osteotomy as a treatment for severe adult spinal deformities: Case series with initial clinical and radiographic outcomes. J. Neurosurg. Spine.

[10] Liu X., Yuan S., Tian Y., Wang L., Zheng Y., Li J. (2015). Expanded eggshell procedure combined with closing-opening technique (a modified vertebral column resection) for the treatment of thoracic and thoracolumbar angular kyphosis. J. Neurosurg. Spine.

[11] Deukmedjian A.R., Le T.V., Baaj A.A., Dakwar E., Smith D.A., Uribe J.S. (2012). Anterior longitudinal ligament release using the minimally invasive lateral retroperitoneal transpsoas approach: A cadaveric feasibility study and report of 4 clinical cases. J. Neurosurg. Spine.

[12] Uribe J.S., Smith D.A., Dakwar E., Baaj A.A., Mundis G.M., Turner A.W., Cornwall G.B., Akbarnia B.A. (2012). Lordosis restoration after anterior longitudinal ligament release and placement of lateral hyperlordotic interbody cages during the minimally invasive lateral transpsoas approach: A radiographic study in cadavers. J. Neurosurg. Spine.

[13] Buell T.J., Nguyen J.H., Mazur M.D., Mullin J.P., Garces J., Taylor D.G., Yen C.P., Shaffrey M.E., Shaffrey C.I., Smith J.S. (2018). Radiographic outcome and complications after single-level lumbar extended pedicle subtraction osteotomy for fixed sagittal malalignment: A retrospective analysis of 55 adult spinal deformity patients with a minimum 2-year follow-up. J. Neurosurg. Spine.

[14] Jain A., Hassanzadeh H., Puvanesarajah V., Klineberg E.O., Sciubba D.M., Kelly M.P., Hamilton D.K., Lafage V., Buckland A.J., Passias P.G. (2017). Incidence of perioperative medical complications and mortality among elderly patients undergoing surgery for spinal deformity: Analysis of 3519 patients. J. Neurosurg. Spine.

[15] Scheer J.K., Smith J.S., Schwab F., Lafage V., Shaffrey C.I., Bess S., Daniels A.H., Hart R.A., Protopsaltis T.S., Mundis G.M. (2017). Development of a preoperative predictive model for major complications following adult spinal deformity surgery. J. Neurosurg. Spine.

[16] Moore B.J., White S., Washington R., Coenen N., Elixhauser A. (2017). Identifying increased risk of readmission and in-hospital mortality using hospital administrative data: The AHRQ elixhauser comorbidity index. Med. Care.

[17] Quan H., Sundararajan V., Halfon P., Fong A., Burnand B., Luthi J.C., Saunders L.D., Beck C.A., Feasby T.E., Ghali W.A. (2005). Coding algorithms for defining comorbidities in ICD-9-CM and ICD-10 administrative data. Med. Care.

[18] Johnson S.R., Tomlinson G.A., Hawker G.A., Granton J.T., Feldman B.M. (2018). Propensity score methods for bias reduction in observational studies of treatment effect. Rheum. Dis. Clin. N. Am..

[19] Lunt M. (2014). Selecting an appropriate caliper can be essential for achieving good balance with propensity score matching. Am. J. Epidemiol..

[20] Fu W.J., Carroll R.J., Wang S. (2005). Estimating misclassification error with small samples via bootstrap cross-validation. Bioinformatics.

[21] Cheng C.L., Kao Y.H., Lin S.J., Lee C.H., Lai M.L. (2011). Validation of the National Health Insurance research database with ischemic stroke cases in Taiwan. Pharmacoepidemiol. Drug Saf..

[22] Lash T.L., Fox M.P., MacLehose R.F., Maldonado G., McCandless L.C., Greenland S. (2014). Good practices for quantitative bias analysis. Int. J. Epidemiol..

[23] Kado D.M., Browner W.S., Palermo L., Nevitt M.C., Genant H.K., Cummings S.R. (1999). Vertebral fractures and mortality in older women: A prospective study. study of osteoporotic fractures research group. Arch. Intern. Med..

[24] Kado D.M., Huang M.H., Karlamangla A.S., Barrett-Connor E., Greendale G.A. (2004). Hyperkyphotic posture predicts mortality in older community-dwelling men and women: A prospective study. J. Am. Geriatr. Soc..

[25] Kado D.M., Lui L.Y., Ensrud K.E., Fink H.A., Karlamangla A.S., Cummings S.R., Study of Osteoporotic F. (2009). Hyperkyphosis predicts mortality independent of vertebral osteoporosis in older women. Ann. Intern. Med..

[26] Worley N., Marascalchi B., Jalai C.M., Yang S., Diebo B., Vira S., Boniello A., Lafage V., Passias P.G. (2016). Predictors of inpatient morbidity and mortality in adult spinal deformity surgery. Eur. Spine J..

[27] Street J.T., Lenehan B.J., DiPaola C.P., Boyd M.D., Kwon B.K., Paquette S.J., Dvorak M.F., Rampersaud Y.R., Fisher C.G. (2012). Morbidity and mortality of major adult spinal surgery. A prospective cohort analysis of 942 consecutive patients. Spine J..

[28] McCarthy I., O’Brien M., Ames C., Robinson C., Errico T., Polly D.W., Hostin R., International Spine Study G. (2014). Incremental cost-effectiveness of adult spinal deformity surgery: Observed quality-adjusted life years with surgery compared with predicted quality-adjusted life years without surgery. Neurosurg. Focus.

[29] Yoshida G., Boissiere L., Larrieu D., Bourghli A., Vital J.M., Gille O., Pointillart V., Challier V., Mariey R., Pellise F. (2017). Advantages and disadvantages of adult spinal deformity surgery and its impact on health-related quality of life. Spine.

[30] Riley M.S., Bridwell K.H., Lenke L.G., Dalton J., Kelly M.P. (2018). Health-related quality of life outcomes in complex adult spinal deformity surgery. J. Neurosurg. Spine.

[31] Haque R.M., Mundis G.M., Ahmed Y., El Ahmadieh T.Y., Wang M.Y., Mummaneni P.V., Uribe J.S., Okonkwo D.O., Eastlack R.K., Anand N. (2014). Comparison of radiographic results after minimally invasive, hybrid, and open surgery for adult spinal deformity: A multicenter study of 184 patients. Neurosurg. Focus.

[32] Park P., Wang M.Y., Lafage V., Nguyen S., Ziewacz J., Okonkwo D.O., Uribe J.S., Eastlack R.K., Anand N., Haque R. (2015). Comparison of two minimally invasive surgery strategies to treat adult spinal deformity. J. Neurosurg. Spine.

[33] Than K.D., Mummaneni P.V., Bridges K.J., Tran S., Park P., Chou D., La Marca F., Uribe J.S., Vogel T.D., Nunley P.D. (2017). Complication rates associated with open versus percutaneous pedicle screw instrumentation among patients undergoing minimally invasive interbody fusion for adult spinal deformity. Neurosurg. Focus.

[34] Wang M.Y., Vasudevan R., Mindea S.A. (2014). Minimally invasive lateral interbody fusion for the treatment of rostral adjacent-segment lumbar degenerative stenosis without supplemental pedicle screw fixation. J. Neurosurg. Spine.

[35] Patel A.A., Zfass-Mendez M., Lebwohl N.H., Wang M.Y., Green B.A., Levi A.D., Vanni S., Williams S.K. (2015). Minimally invasive versus open lumbar fusion: A comparison of blood loss, surgical complications, and hospital course. Iowa Orthop. J..

[36] Smith Z.A., Fessler R.G. (2012). Paradigm changes in spine surgery: Evolution of minimally invasive techniques. Nat. Rev. Neurol..

[37] Wu J.C., Chen Y.C., Liu L., Chen T.J., Huang W.C., Cheng H., Tung-Ping S. (2012). Increased risk of stroke after spinal cord injury: A nationwide 4-year follow-up cohort study. Neurology.

[38] Chung W.F., Liu S.W., Huang L.C., Chang H.K., Wu J.C., Chen L.F., Chen Y.C., Huang W.C., Cheng H., Lo S.S. (2017). Serious dysphagia following anterior cervical discectomy and fusion: Long-term incidence in a national cohort. J. Neurosurg. Sci..

[39] Chen L.F., Tu T.H., Chen Y.C., Wu J.C., Chang P.Y., Liu L., Huang W.C., Lo S.S., Cheng H. (2016). Risk of spinal cord injury in patients with cervical spondylotic myelopathy and ossification of posterior longitudinal ligament: A national cohort study. Neurosurg. Focus.

[40] Huang L.C., Chung W.F., Liu S.W., Chang P.Y., Chen L.F., Wu J.C., Chen Y.C., Huang W.C., Liu L., Cheng H. (2015). Lower risk of stroke after deformity surgery: Long term benefit demonstrated by a National Cohort study. Int. J. Environ. Res. Public Health.

[41] Elixhauser Comorbidity Software, Version 3.7. https://www.hcup-us.ahrq.gov/toolssoftware/comorbidity/comorbidity.jsp#overview.

[42] Charlson Comorbidities Index. http://mchp-appserv.cpe.umanitoba.ca/viewConcept.php?conceptID=1098.

